# Book Review: Social Ecology in the Digital Age: Solving Complex Problems in a Globalized World

**DOI:** 10.3389/fsoc.2019.00027

**Published:** 2019-04-18

**Authors:** Calvin P. Tribby, Lilian G. Perez, David Berrigan

**Affiliations:** Health Behaviors Research Branch, Behavioral Research Program, Division of Cancer Control and Population Sciences, National Cancer Institute, Bethesda, MD, United States

**Keywords:** social ecology, multi-level research, digital health, trans-disciplinary, environmental justice (or climate justice)

This book examines the history, scientific value, and unifying framework of social ecology for addressing community and global problems in the wake of dramatic technological advancements. Social ecology blends theories and approaches from multiple disciplines, such as environmental psychology and sociology, to facilitate interdisciplinary multilevel research for the development and maintenance of health-promoting behaviors and environments (Stokols, 1992). Multilevel research strategies measure and model several levels of influence (e.g., individual, social, environment, digital) and their interactions to study and intervene on complex phenomena (Hall et al., 2018). Dr. Stokols weaves his personal and professional journey from graduate school into the professoriate at the University of California, Irvine into this extended argument for the distinct importance and power of social ecology. In Dr. Stokols's argument, the digital sphere is necessary to enhance social ecology research, both as an addition to the theoretical framework and as a subject of study to tackle today's urgent and complex global issues. Existing frameworks have mostly neglected the digital sphere, yet digital technologies have drastically altered social connections, the built environment (e.g., app-based transportation modes), economic functions (e.g., how individuals shop, travel, work), and the natural environment (e.g., climate change). Inclusion of the digital sphere is necessary to fully capture people's changing relationships with their natural, sociocultural, and built environments. This ambitious effort to define and summarize an entire discipline provides a fascinating argument for the utility of social ecology and offers an engaging personal journey through academia. This personal journey into a relatively new field was risky and offers a paragon to guide scholars similarly confronted with the decision of moving into interdisciplinary programs.

Chapter 1 covers Dr. Stokols's personal journey from the discipline of psychology into social ecology and Chapter 2 details the origins of social ecology. It starts with the history of ecology and how ecological concepts were transferred from environmental systems research to the study of how social and environmental contexts affected people and reviews the theoretical beginning of socio-ecological models. Chapter 3 serves as the formal introduction to social ecology principles, which takes an “interdisciplinary, multilevel, multimethod, systems-oriented, translational approach” to research (p. 66). Chapter 4, Rise of the Internet, is where this book outlines the relevance of social ecology in the digital age. This chapter summarizes the transition from predigital times and reviews examples of changes in built environments and social behavior due to digital technologies. Dr. Stokols then defines the distinct components of cyberspace (digital communications and virtual spaces) as episodic communications (e.g., text messages and email), virtual behavior settings (e.g., Facebook and Amazon), and virtual communities (e.g., online gaming and support groups). The links between these cyberspace components and the physical world form new units of analysis to examine the diverse impacts of the cybersphere.

Chapters 5–8 provide domain-specific applications of social ecology principles in public health, complex social problems, global environmental changes, and sustainable communities. These chapters cover a diverse range of problems and the motivating examples make clear the advantages of using social ecology and the cybersphere. For example, Chapter 6 presents a case study of how the cybersphere can both add to the theoretical framework and be a subject of study. The Digital Youth Network (DYN) is an ecological intervention that leverages a multilevel structure, consisting of virtual and place-based learning across classroom, home, and after-school settings, to reduce the digital divide among disadvantaged public school students (Barron et al., 2014). The DYN further identified community resources to support the digital literacy programming outside of the school and identified neighborhoods needing further investment. This is contrasted in the chapter with an unsuccessful digital literacy intervention in Los Angeles that was only curriculum-based and did not include the social, such as family or after-school peer programs, or environmental supports, such as neighborhood sites to access free WiFi. Chapter 7, Managing Global Environmental Change, is a very detailed effort to describe the history and impacts of human-caused greenhouse gases. Given the US government's recent scientific report (Reidmiller et al., 2018) detailing the predicted future consequences of climate change on human health, food production, and global economies, especially in the most vulnerable communities, social ecology may be a key field to develop and integrate a blueprint of how multiple sectors can work together to address complex global problems. Dr. Stokols reviews actions at various levels (e.g., individual, corporate) that may help address climate change, yet the role of the cybersphere and technology companies is a frontier. For example, do social media contribute to social inclusion and strengthen the collective response to climate change or to social isolation and the spread of “fake news” and climate change denial?

Chapter 9 concludes with training the next generation of social ecologists. Dr. Stokols suggests that social ecology, like public health, is fundamentally a moral endeavor and that training should include instilling a moral code and transformative agenda in its trainees and practice. The translational approach to science in social ecology encourages research to improve environmental and public health and reduce societal inequities. To achieve these aims, transdisciplinary practice and transdisciplinary mentors in social ecology will help foster students' dedication to addressing pressing environmental and social challenges. However, there are challenges with training transdisciplinary scholars, as it requires developing a personal transdisciplinary orientation, based on values, beliefs, attitudes, and behaviors favorable for integration and collaboration. More evidence is needed into how researchers who do not possess or are uninterested in cultivating these personal attributes may be included into the transdisciplinary research paradigm.

Despite the emphatically transdisciplinary approach adopted by Dr. Stokols and by social ecology, renewed attention to several key partners could enhance the influence of social ecology in the digital age on efforts to address the challenges laid out in this book. For example, although the text provides examples of environmental justice research, the field could benefit from greater integration of environmental justice expertise into its conceptual frameworks to better address disadvantaged populations and disparities in exposures and outcomes. Additionally, the discipline could benefit from a more thorough integration of economic and business perspectives by fostering partnerships with economists and the technology sector. Social and policy changes to address environmental and health threats may be more convincing to the public and policy makers and successful if the impacts of such threats to country- and global-level economics are explicitly considered and communicated. Further, the technology sector plays a critical role in the type of information/news people get about global problems and this can influence how communities and decision-makers respond. Finally, community engagement in these transdisciplinary efforts will be critical for informing solutions, as community members can provide valuable insight into the potential (intended and unintended) consequences of such efforts.

We found this book highly readable and thought provoking. It would be an excellent text for an advanced undergraduate course or an early graduate-level seminar in a diverse array of disciplines, such as public health, geography, or sociology, especially if supplemented with further material on how to conduct social ecology research. Overall, this text provides a historical accounting of the evolution of social ecology and its transdisciplinary approach and cements Dr. Stokols's role as a key leader in articulating a unifying vision for social ecology in the digital age.

## Author Contributions

All authors listed have made a substantial, direct and intellectual contribution to the work, and approved it for publication.

### Conflict of Interest Statement

The authors declare that the research was conducted in the absence of any commercial or financial relationships that could be construed as a potential conflict of interest.
